# Hypoxia-Responsive Azobenzene-Linked Hyaluronate Dot Particles for Photodynamic Tumor Therapy

**DOI:** 10.3390/pharmaceutics14050928

**Published:** 2022-04-24

**Authors:** Sohyeon Lee, Yoonyoung Kim, Eun Seong Lee

**Affiliations:** 1 Department of Biotechnology, The Catholic University of Korea, 43 Jibong-ro, Bucheon-si 14662, Gyeonggi-do, Korea; sohye0n22@naver.com (S.L.); rladbsdud727@naver.com (Y.K.); 2 Department of Biomedical-Chemical Engineering, The Catholic University of Korea, 43 Jibong-ro, Bucheon-si 14662, Gyeonggi-do, Korea

**Keywords:** hypoxia-sensitive, hyaluronate dot particles, ultra-small size, chlorin e6, photodynamic tumor therapy

## Abstract

In this study, we developed ultra-small hyaluronate dot particles that selectively release phototoxic drugs into a hypoxic tumor microenvironment. Here, the water-soluble hyaluronate dot (dHA) was covalently conjugated with 4,4′-azodianiline (Azo, as a hypoxia-sensitive linker) and Ce6 (as a photodynamic antitumor agent), producing dHA particles with cleavable Azo bond and Ce6 (dHA-Azo-Ce6). Importantly, the inactive Ce6 (self-quenched state) in the dHA-Azo-Ce6 particles was switched to the active Ce6 (dequenched state) via the Azo linker (–N=N–) cleavage in a hypoxic environment. In vitro studies using hypoxia-induced HeLa cells (treated with CoCl_2_) revealed that the dHA-Azo-Ce6 particle enhanced photodynamic antitumor inhibition, suggesting its potential as an antitumor drug candidate in response to tumor hypoxia.

## 1. Introduction

Abnormal proliferation of tumor cells, multifactorial mutagenic cell metabolism, and lack of oxygen and nutrients cause tumor hypoxia, which not only worsens the prognosis of tumor patients [1,2,3,4,5], but also decreases the antitumor efficiency of chemotherapy. Importantly, hypoxia with the limited microcirculation and deterioration of diffusion conditions decreases drug concentration in tumor cells and acts as a hurdle in tumor therapy, structurally and functionally [1,2,4,5,6]. Therefore, it is essential to develop an environmentally responsive drug delivery system, based on functional polymers, to overcome in vivo tumor hypoxia [6,7,8,9,10]. Prodrug-based drug-carrying nano-sized vehicles that include a smart reactive chemical group that selectively responds to a specific tumor environment are expected to increase drug sensitivity to tumor cells [7,8,10,11]. In addition to establishing such functionality, it is also necessary to design drug particles with optimized size to promote penetration into hypoxia tumors. Results of recent studies suggest that ultra-small particles (approximately 10 nm in size) can have higher tumor penetration than nanoparticles of 100–200 nm in size [12,13,14,15].

**Figure 1:**
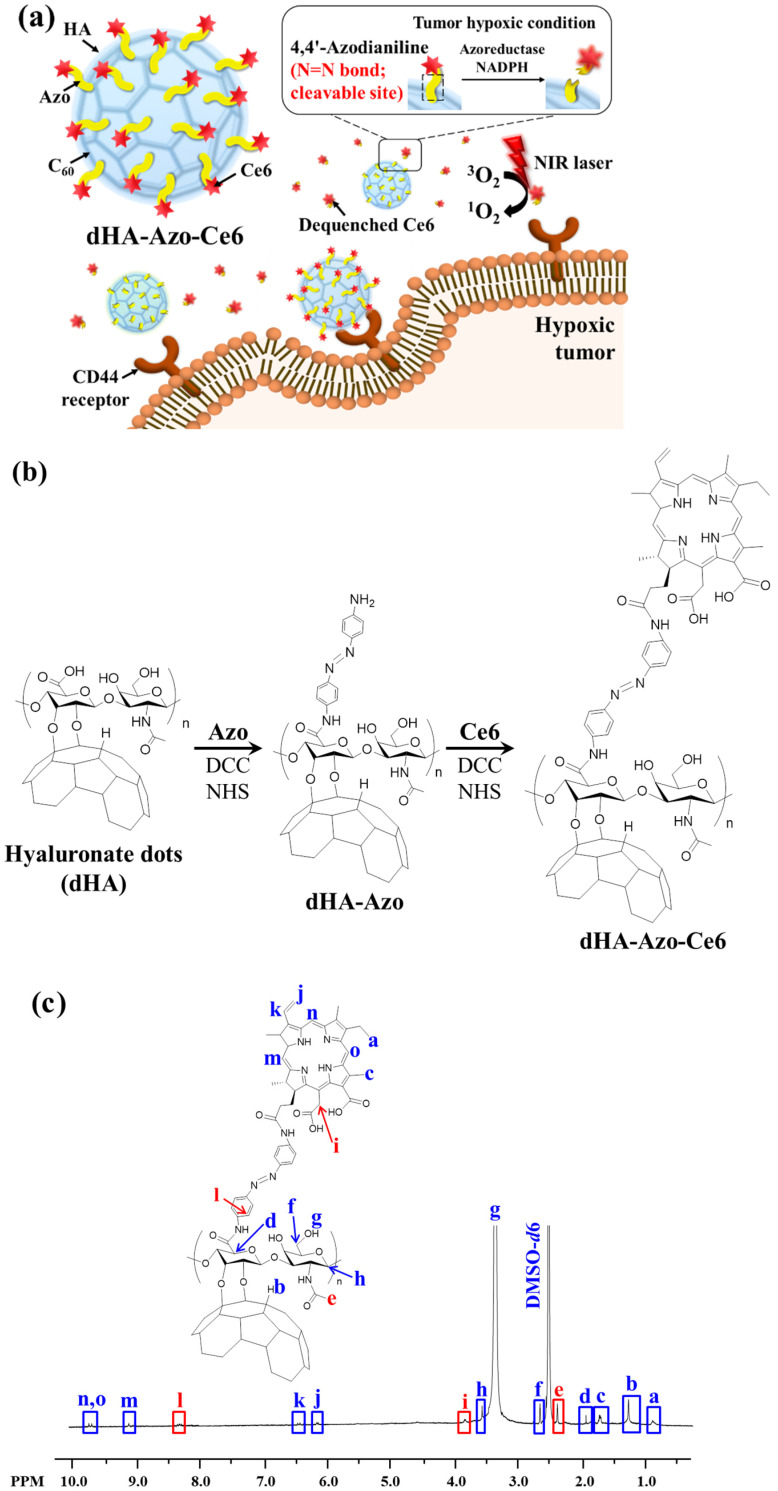

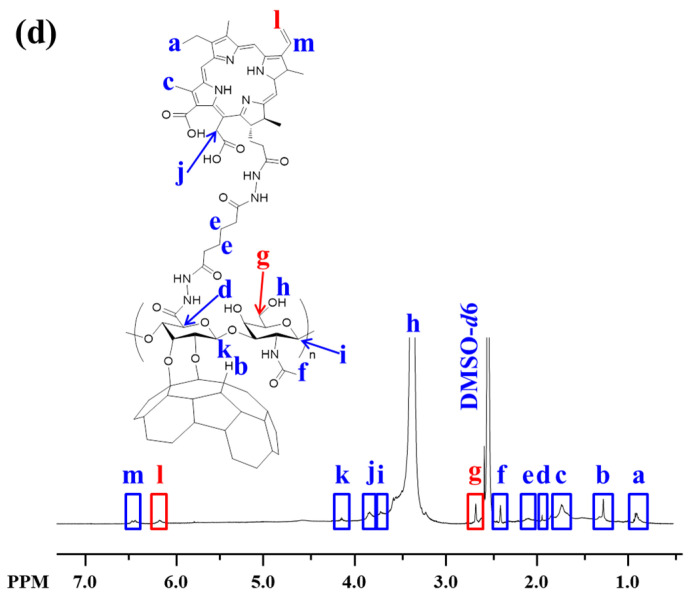
(**a**) Schematic illustration of dHA-Azo-Ce6. (**b**) Synthesis scheme of dHA-Azo-Ce6. (**c**) ^1^H-NMR peaks of dHA-Azo-Ce6. (**d**) ^1^H-NMR peaks of dHA-Ce6.

In this study we report ultra-small particles with a hypoxia-responsive chemical linker for efficient treatment of hypoxia tumors. In a previous study, our group fabricated hyaluronic acid (HA) dots (dHA, 5~10 nm size) [15,16,17,18] by conjugating biocompatible HA (M_n_ = 3.7 kDa) [15,16,17,18] with buckyball-like C_60_. Subsequently, we prepared dHA with azobenzene (Azo, as a chemical linker responding to tumor hypoxia condition) [7,19,20,21,22] and chlorin e6 (Ce6, a photoactive model drug) [17,23,24,25]. The resulting dHA-Azo-Ce6 is expected to efficiently penetrate the tumor tissue, and the Azo linker would be degraded by nicotinamide adenine dinucleotide phosphate (NADPH) and azo-reductase, which are abundant in the hypoxia condition [6,9], finally producing free photoactive/tumor-toxic Ce6 from the dots (Figure 1a). We preferentially investigated the in vitro antitumor activity of dHA-Azo-Ce6 particle to confirm its potential as an antitumor drug delivery system for treating hypoxia tumors.

## 2. Materials and Methods

### 2.1. Materials

Hyaluronic acid (HA, M_n_ = 3.7 kDa), toluene, *N*,*N*′-dicyclohexylcarbodiimide (DCC), dimethyl sulfoxide (DMSO), *N*-hydroxysuccinimide (NHS), lithium hydroxide (LiOH), triethylamine (TEA), sodium dithionite (Na_2_S_2_O_4_), 4′,6-diamidino-2-phenylindole dihydrochloride (DAPI), adipic acid dihydrazide (ADH), and 9,10-dimethylanthracene (DMA) were bought from Sigma-Aldrich (St. Louis, MO, USA). 4,4′-Azodianiline (Azo) was purchased from AA blocks Inc. (San Diego, CA, USA). C_60_ was provided from NanoLab Inc. (Waltham, MA, USA). Cobalt (II) chloride hexahydrate (CoCl_2_) was purchased from Tokyo Chemical Industry (Tokyo, Japan). Chlorin e6 (Ce6) was provided from Frontier Scientific Inc. (Logan, UT, USA). Bovine calf serum (BCS), penicillin, Dulbecco’s modified eagle medium (DMEM), phosphate-buffered saline (PBS, pH 7.4), streptomycin, fetal bovine serum (FBS), trypsin, and ethylene diamine tetraacetic acid (EDTA) were bought from Welgene Inc. (Seoul, Korea). HIF-1α ELISA kit was purchased from Thermo Fisher Scientific, Inc. (Waltham, MA, USA). Wheat germ agglutinin alexa fluor^®^ 488 conjugate (WGA-Alexa fluor^®^ 488) was bought from Life Technologies (Carlsbad, CA, USA). Cell counting kit-8 (CCK-8) was provided from Dojindo Molecular Technologies Inc. (Rockville, MD, USA).

### 2.2. Synthesis of dHA-Azo-Ce6

As described in our previous reports [15,16,17,18], HA (1.2 g) reacted with C_60_ (160 mg) in DMSO (20 mL)/toluene (20 mL) containing LiOH (as a catalyst) at 25 °C for 3 days, producing dHA. Subsequently, our dHA (20 mg, M_n_ ~ 14 kDa) [15,16,17,18] reacted with excess Azo (50 mg) in DMSO (20 mL) containing DCC (25 mg), NHS (30 mg), and TEA (1 mL) at 25 °C for 3 days (Figure 1b), producing dHA with Azo (dHA-Azo). The resulting solution was purified using a Spectra/Pro^®^ MWCO 10 kDa membrane tube, and then lyophilized [15,16,17,18]. Next, dHA-Azo (200 mg) reacted with Ce6 [100 mg, pre-activated using DCC (35 mg), NHS (40 mg), and TEA (1 mL) in DMSO at 25 °C for 4 h] in DMSO (20 mL) at 25 °C for 3 days, producing dHA-Azo-Ce6 aggregates. The conjugation ratio of Azo and Ce6 in dHA-Azo-Ce6 were analyzed using a ^1^H NMR (Bruker Advance III 500 MHz, Billerica, MA, USA) [15,16,17,19,23]. In addition, we prepared dHA with Ce6 (dHA-Ce6) as a control group (without Azo) after directly coupling dHA [200 mg, pre-activated in DMSO (15 mL) containing DCC (20 mg) and NHS (25 mg) at 25 °C for 1 day] [15,16,17] to Ce6 [20 mg, pre-activated in DMSO (15 mL) containing ADH (60 mg), DCC (20 mg), NHS (25 mg), and TEA (1 mL) at 25 °C for 4 h] [15,16,17] in DMSO 25 °C for 2 days. The solution was purified using a Spectra/Pro^®^ MWCO 10 kDa membrane tube against fresh DMSO for 2 days and then deionized water for 2 days, and then lyophilized. The conjugation ratio of Ce6 in dHA-Ce6 were analyzed using a ^1^H NMR (Bruker Advance III 500 MHz, Billerica, MA, USA) [15,16,17].

### 2.3. Characterization of dHA-Azo-Ce6

To analyze the reactivity of the Azo linker and the change in physicochemical properties of dHA-Azo-Ce6 due to decomposition of the Azo linker [7,19,20,21,22], we investigated the properties of dHA-Azo-Ce6 under abundant azo-reductase condition. First, the morphologies of each sample (0.1 mg/mL, in 150 mM PBS pH 7.4) treated with or without azo-reductase (10 mM sodium dithionite, similar to hypoxia condition) [26] for 1 h were evaluated using a Talos L120C transmission electron microscope (TEM, Thermo Scientific Inc., Waltham, MA, USA) [15,17,27,28]. The particle size and zeta potential of each sample (0.1 mg/mL, in 150 mM PBS pH 7.4) treated with or without azo-reductase (10 mM Na_2_S_2_O_4_) for 1 h were analyzed using Zetasizer 3000 (Malvern Instruments, Malvern, UK). The ultraviolet/visible (UV/Vis) absorbance of each sample (equivalent Ce6 10 μg/mL) in DMSO was monitored using a NanoDrop™ 2000/2000c spectrophotometer (Thermo Fisher Scientific Inc., Waltham, MA, USA) [17].

The cumulative Ce6 release from the particles immersed in a dialysis membrane bag (Spectra/Por^®^ MWCO 5 K) was monitored under PBS (150 mM, pH 7.4) with or without azo-reductase (10 mM Na_2_S_2_O_4_) for 24 h using a microplate reader (Bio-Tek, Winooski, VT, USA) at *λ_ex_* of 450 nm and *λ_em_* of 670 nm [29,30,31]. Here, the particles in the dialysis membrane bag were incubated in a shaking water bath (100 rpm) at 37 °C. The solution coming out of the dialysis membrane bag was sampled at each given time and then analyzed [29,30,31].

The light emission of each sample (equivalent to Ce6 10 µg/mL) at *λ_ex_* of 400 nm and *λ_em_* of 600–750 nm was monitored using an RF-5301PC spectrofluorometer (RF-5301, Shimadzu, Kyoto, Japan) in PBS (150 mM, pH 7.4) with or without azo-reductase (10 mM Na_2_S_2_O_4_) [31].

The singlet oxygen generation from each sample (equivalent to Ce6 10 µg/mL) treated with or without azo-reductase (10 mM sodium dithionite) [17,24,32,33] for 1 h was evaluated using florescent 9,10-dimethylanthracene (DMA) [17,32,33]. Here, DMA whose fluorescence decreases when singlet oxygen is present was added to the sample and the resulting solution was irradiated for 10 min at a light intensity of 5.2 mW/cm^2^ using a 670 nm laser source. The generation of singlet oxygen was measured using the DMA fluorescence intensity change (F_f_–F_s_) at *λ_ex_* of 360 nm and *λ_em_* of 380–540 nm using an RF-5301PC spectrofluorometer (RF-5301, Shimadzu, Kyoto, Japan), where F_f_ is the fluorescence intensity of intact DMA and F_s_ is the fluorescence intensity of each sample solution [32,33].

### 2.4. Cell Culture

Human cervical carcinoma HeLa cells and mouse fibroblast NIH3T3 cells were provided from Korean Cell Line Bank (Seoul, Korea). HeLa cells were cultured in the DMEM/10% FBS medium containing 1% penicillin-streptomycin at 37 °C. Mouse fibroblast NIH3T3 cells were cultured in the DMEM/10% BCS medium containing 1% penicillin-streptomycin at 37 °C [15,16,18,34].

### 2.5. HIF-1α Expression

We added 25 mM CoCl_2_ to the cells and adjusted the concentration of CoCl_2_ to 100 µM in the cell culture medium to induce hypoxia environment in cells. The cells treated with CoCl_2_ for 24 h at 37 °C were washed with fresh PBS (150 mM) and then evaluated using a HIF-1α ELISA kit. In addition, the cells pretreated with 25 mM CoCl_2_ were further incubated with each sample (equivalent Ce6 10 μg/mL) for 2 h at 37 °C. These cells were analyzed to determine the effect of each sample on the HIF-1α expression [35,36].

### 2.6. In Vitro Cellular Uptake

The cells pretreated with 25 mM CoCl_2_ for 24 h at 37 °C and those without pretreatment were incubated with each sample (equivalent Ce6 10 μg/mL) for 2 h and then analyzed using a FACS Calibur^TM^ flow cytometer (Becton Dickinson, Franklin Lakes, NJ, USA) [15,16,17,31]. Furthermore, the treated cells were stained using DAPI (for nucleus) [15,16,17,31] and WGA-Alexa fluor^®^488 (for cellular membrane) [15,16,17,31] and then evaluated using a confocal laser scanning microscope (LSM710, Carl Zeiss, Oberkochen, Germany). In addition, the treated cells were also visualized using a Nikon microscope equipped with a visible and near-infrared (VNIR) hyperspectral camera (CytoViva, Auburn, AL, USA) [18].

### 2.7. In Vitro Phototoxicity

The cells pretreated with 25 mM CoCl_2_ for 24 h at 37 °C and those without pretreatment were incubated with each sample (equivalent Ce6 10 μg/mL) for 2 h at 37 °C. The treated cells were washed with fresh PBS (150 mM, pH 7.4) and then irradiated using for 10 min at a light intensity of 5.2 mW/cm^2^ using a 670 nm laser source. The irradiated cells were further incubated in fresh medium without the sample for 12 h at 37 °C. The in vitro cell viability of the treated cells was evaluated using a CCK-8 assay. In addition, the cytotoxicity of dHA-Azo-Ce6 and dHA-Ce6 without light irradiation was evaluated after treatment for 24 h [15,17,31].

### 2.8. Statistical Evaluation

All the results obtained in the experiment were analyzed using a Student’s *t*-test or analysis of variance (ANOVA) at a significance level of *p* < 0.01 (**) [15,16,17,33,34].

## 3. Results and Discussion

### 3.1. Synthesis of dHA-Azo-Ce6 Particles

To fabricate hypoxia-responsive dHA-Azo-Ce6 particles for photodynamic tumor therapy (Figure 1a), we conjugated dHA (M_n_ ~ 14 kDa, prepared after coupling HA with C_60_) [15,16,17,18] with Azo using DCC, NHS, and TEA in DMSO. A phototoxic model drug (Ce6) was then covalently attached to the dHA particles using DCC and NHS in DMSO (Figure 1b). The resulting dHA-Azo-Ce6 particles were analyzed using ^1^H-NMR, and the conjugation molar ratios of Azo and Ce6 to dHA were calculated after evaluating an integration ratio of peaks from δ 8.37 ppm (-CH of Azo), δ 3.83 ppm (-CH_2_ of Ce6), and δ 2.36 ppm (-CH_3_ of dHA) (Figure 1c). It was found that the average conjugation molar ratios of Azo and Ce6 to dHA were 24 and 23, respectively. Next, dHA-Ce6 particles were prepared as a hypoxia-nonresponsive group. The conjugation molar ratio of Ce6 to dHA was calculated after evaluating an integration ratio of peaks from 6.16 ppm (-CH_2_ of Ce6) and δ 2.64 ppm (-CH_2_ of dHA) (Figure 1d). The average conjugation molar ratio of Ce6 to dHA was 22.

### 3.2. Characterization of dHA-Azo-Ce6 Particles

**Figure 2:**
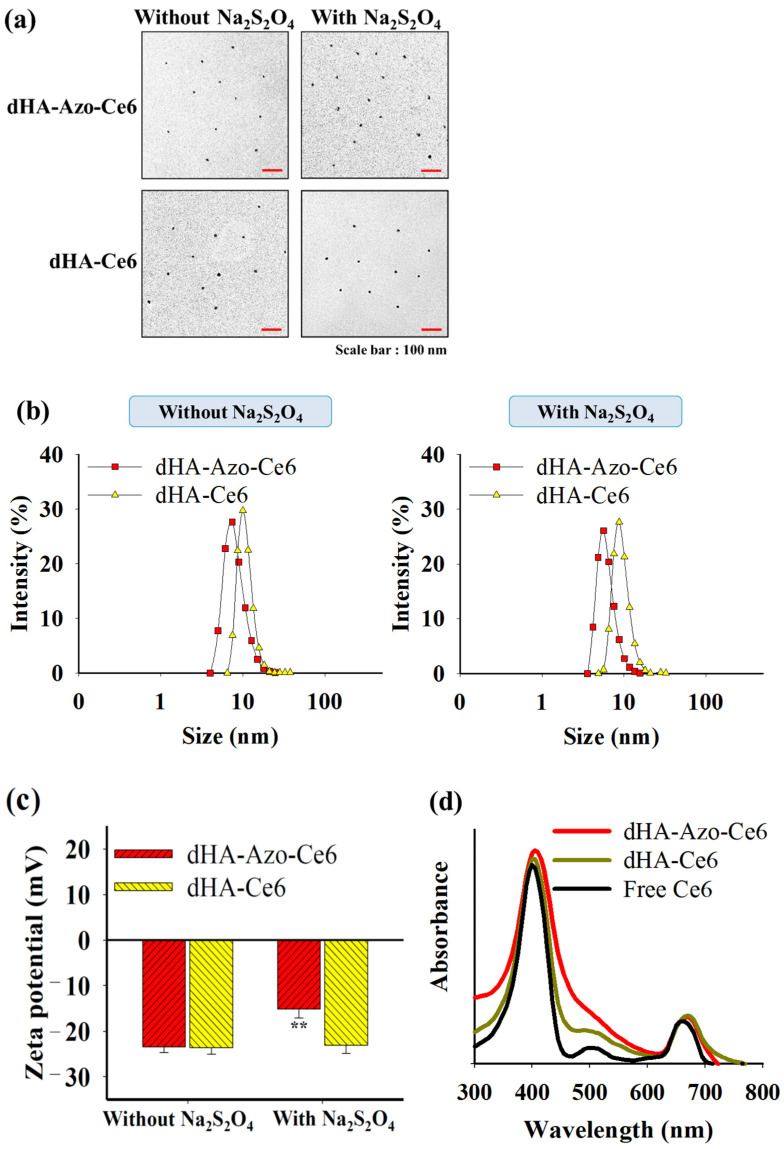
(**a**) TEM images of dHA-Azo-Ce6 and dHA-Ce6 with or without azo-reductase (Na_2_S_2_O_4_) treatment. (**b**) Particle size distribution and (**c**) zeta potential values of dHA-Azo-Ce6 and dHA-Ce6 with or without azo-reductase (Na_2_S_2_O_4_) treatment (n = 3, as multiple experiments, ** *p* < 0.01 compared to dHA-Ce6). (**d**) UV/Vis spectra of each sample (equivalent Ce6 10 μg/mL) and free Ce6 (10 μg/mL) in DMSO.

Figure 2a,b show that the dHA particles have the shape of an ultra-small dot (4–12 nm in diameter). There were no morphological or particle size changes in the dHA particles that were treated with or without Na_2_S_2_O_4_. However, the zeta potential values of the dHA-Azo-Ce6 particles increased from −23.4 mV (without Na_2_S_2_O_4_ treatment) [26] to −15.1 mV (with Na_2_S_2_O_4_ treatment), probably due to the Na_2_S_2_O_4_ (azo-reductase)-induced chemical cleavage of Azo linkers in the dHA particles (Figure 2c). We confirmed that the dHA-Azo-Ce6 particles treated with 10 mM Na_2_S_2_O_4_ for 2 h resulted in the chemical cleavage of Azo followed by the release of Ce6, as indicated by the disappearance of the ^1^H-NMR peaks of Azo and Ce6 (Figure S1). In contrast, the hypoxia-nonresponsive dHA-Ce6 particles with Na_2_S_2_O_4_ treatment exhibited no changes in zeta potential values as compared to those that were left untreated (Figure 2c). In addition, the UV/Vis absorbance spectra of the dHA particles showed the peaks of absorption bands at 400 and between 650 and 670 nm [17], consistent with the typical absorption bands of the photosensitive Ce6 molecule (Figure 2d). Next, we evaluated the cumulative Ce6 release pattern of the dHA particles that were treated with Na_2_S_2_O_4_ and those left untreated.

**Figure 3:**
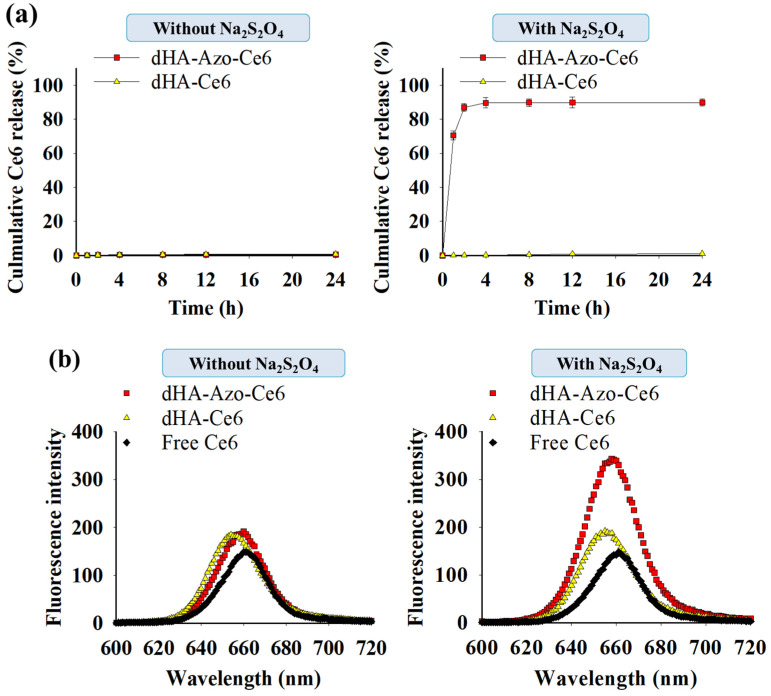

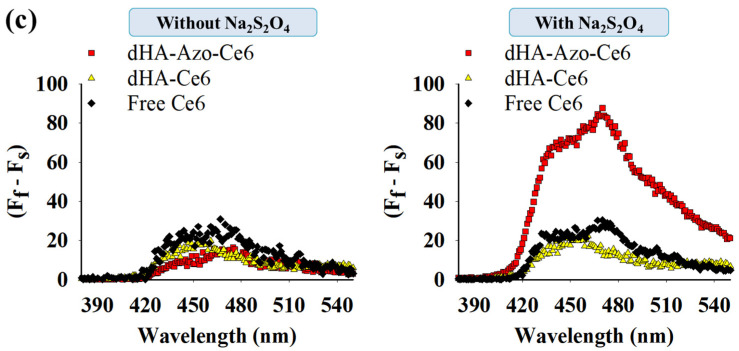
(**a**) Cumulative Ce6 release from dHA-Azo-Ce6 and dHA-Ce6 with or without azo-reductase (Na_2_S_2_O_4_) treatment (n = 3, as multiple experiments). (**b**) The emission spectra (at *λ_ex_* of 400 nm and *λ_em_* of 600–720 nm) of each sample (equivalent Ce6 10 μg/mL) and free Ce6 (10 μg/mL) in PBS (pH 7.4, 150 mM) with or without azo-reductase (Na_2_S_2_O_4_) treatment. (**c**) 9,10-dimethylanthracene (DMA) fluorescence change (at *λ_ex_* 360 nm and *λ_em_* 380–550 nm) of each sample (equivalent Ce6 10 μg/mL) and free Ce6 (10 μg/mL) in PBS (pH 7.4, 150 mM) with or without azo-reductase (Na_2_S_2_O_4_) treatment.

As shown in Figure 3a, the dHA-Azo-Ce6 particles with Na_2_S_2_O_4_ treatment presented rapid Ce6 release (89 wt.%) in 4 h, but the hypoxia-nonresponsive dHA-Ce6 particles did not show Ce6 release. These results indicate that Azo linkers in the dHA-Azo-Ce6 particles were degraded by azo-reductase (Na_2_S_2_O_4_), which highly accelerated the release of Ce6. Figure 3b shows the emission spectra (at *λ_ex_* 400 nm) of the dHA particles with or without Na_2_S_2_O_4_ treatment. It was found that the dHA-Azo-Ce6 particles with Na_2_S_2_O_4_ treatment resulted in the significant increase in fluorescence intensity at 670 nm. This event is probably due to the Ce6 molecules conjugated with the dHA particles (without Na_2_S_2_O_4_ treatment) being in an auto-quenched state (decrease in fluorescence intensity), and the Ce6 molecules released from the dHA particles (with Na_2_S_2_O_4_ treatment) being in a dequenched state (increase in fluorescence intensity) [16]. Similarly, the dHA-Azo-Ce6 particles with Na_2_S_2_O_4_ treatment were effective in generating singlet oxygen under light irradiation, as shown in the increase of DMA fluorescence change (Figure 3c). It appears that Ce6 molecules dissociated from the dHA particles are advantageous for generating singlet oxygen. However, the dHA-Ce6 particles and free Ce6, in the auto-quenched state, exhibited low levels of single oxygen generation under light irradiation.

### 3.3. In Vitro Tumor Inhibition

**Figure 4:**
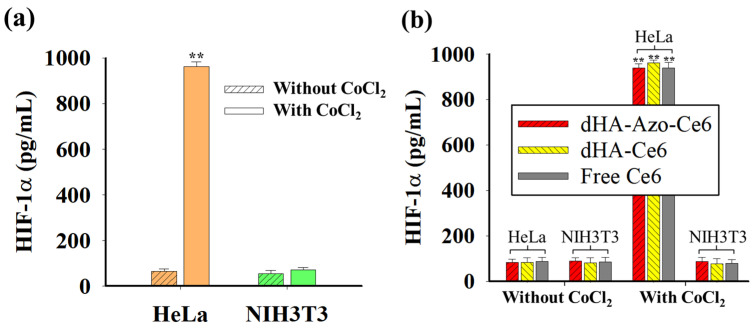
(**a**) HIF-1α expression levels in cells (HeLa and NIH3T3) with or without CoCl_2_ treatment (n = 3, as multiple experiments, ** *p* < 0.01 compared to HeLa cells without CoCl_2_). (**b**) HIF-1α expression levels in HeLa and NIH3T3 cells treated with each sample (10 μg/mL equivalent of Ce6) or free Ce6 (10 μg/mL) before and after CoCl_2_ treatment (n = 7, as multiple experiments, ** *p* < 0.01 compared to HeLa or NIH3T3 cells without CoCl_2_).

Figure 4a shows the hypoxia-inducible factor (HIF)-1α [7,35,36] expression in cells treated with CoCl_2_. It is known that HIF-1α, found in various tumor tissues, promotes the production of erythropoietin and vascular endothelial growth factor to facilitate oxygen transport and helps tumor cells survive under hypoxia environments [7,35,36]. As a result, the treatment of CoCl_2_ (a hypoxia-mimetic agent) resulted in the increased expression of HIF-1α in HeLa cells. However, the normal NIH3T3 cells did not respond to the CoCl_2_ treatment, presenting negligible HIF-1α expression level (Figure 4a). In addition, we investigated whether the particles affect HIF-1α expression in cells. As shown in Figure 4b, none of the samples had any effect on the HIF-1α expression in the cells.

**Figure 5:**
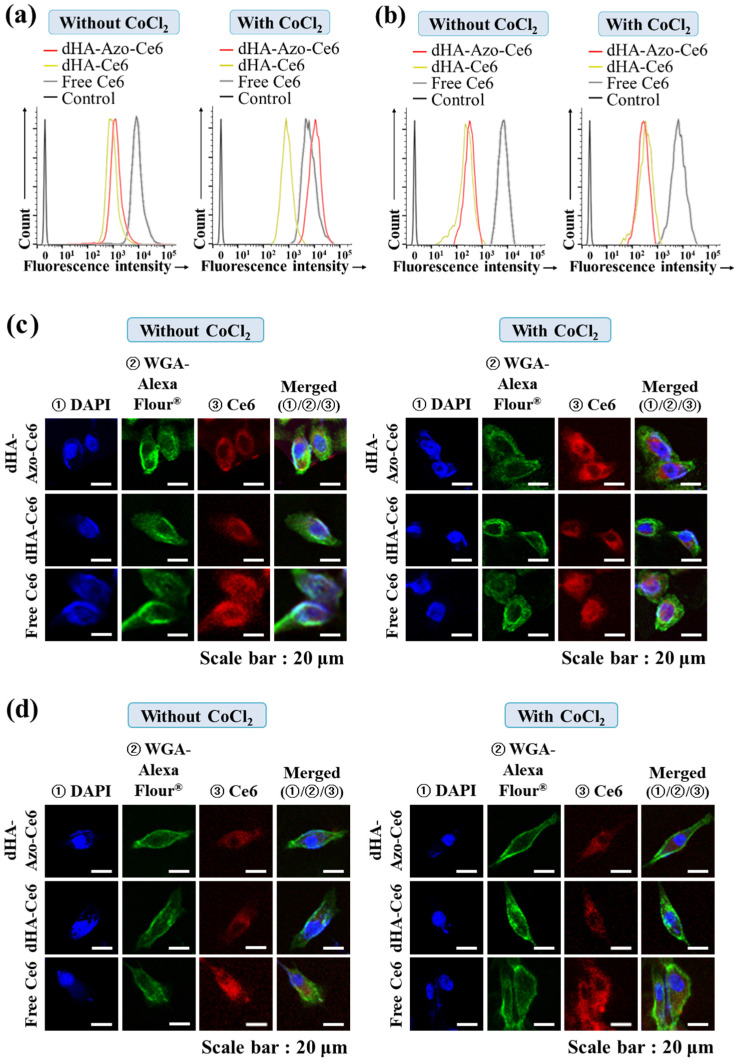

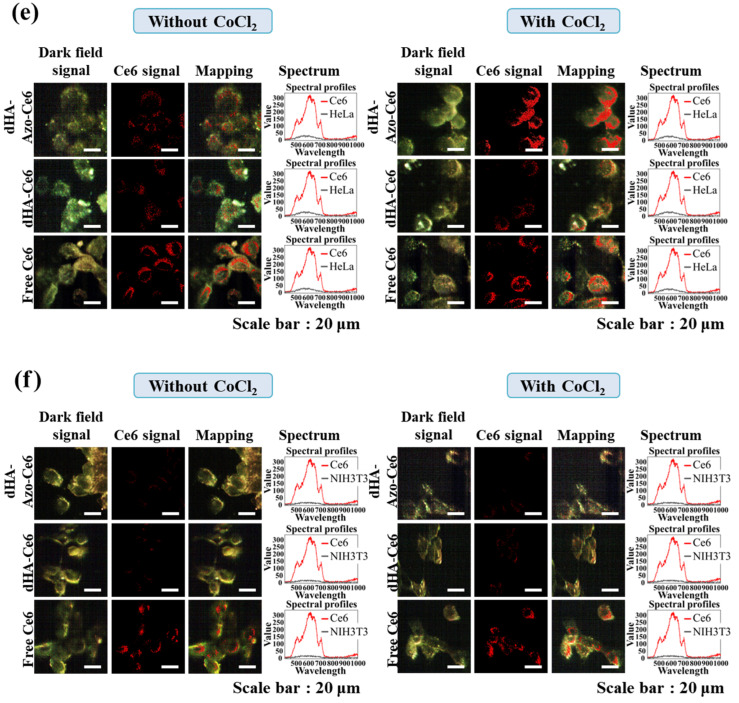
Flow cytometry analysis of (**a**) HeLa and (**b**) NIH3T3 cells treated with each sample (equivalent Ce6 10 μg/mL) or free Ce6 (10 μg/mL) under environment with CoCl_2_ or without CoCl_2_ for 2 h at 37 °C. Confocal images of (**c**) HeLa and (**d**) NIH3T3 cells treated with each sample (equivalent Ce6 10 μg/mL) or free Ce6 (10 μg/mL) under environment with CoCl_2_ or without CoCl_2_ for 2 h at 37 °C. Hyperspectral images of (**e**) HeLa and (**f**) NIH3T3 cells treated with each sample (equivalent Ce6 10 μg/mL) or free Ce6 (10 μg/mL) under environment with CoCl_2_ or without CoCl_2_ for 2 h at 37 °C.

Next, we evaluated the cellular uptake of Ce6 in the CoCl_2_-treated cells (HeLa tumor cells [7,24,35] and normal NIH3T3 cells [36]). Figure 5a shows the increased Ce6 uptake of dHA-Azo-Ce6 particles in the HeLa tumor cells treated with CoCl_2_ [7,24,35]. Under hypoxia conditions [24], the CoCl_2_-treated HeLa cells promoted the degradation of the Azo linker in the dHA-Azo-Ce6 particles, accelerating the Ce6 release (Figure 3a) to the cells. However, the NIH3T3 normal cells showed no difference in Ce6 uptake rate for all sample groups (Figure 5b). Of course, it is thought that some particles were absorbed into the NIH3T3 normal cells by general pinocytosis because the particles (4–12 nm in diameter) were very small.

Similarly, the confocal images (Figure 5c,d) and hyperspectral images (Figure 5e,f) of cells treated with dHA-Azo-Ce6 particles revealed that the dHA-Azo-Ce6 particles enabled a large increase in Ce6 uptake in CoCl_2_–treated HeLa tumor cells compared to untreated HeLa tumor cells and NIH3T3 normal cells.

**Figure 6:**
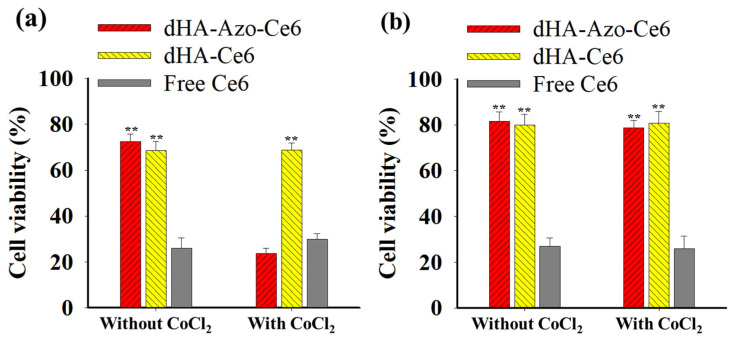
Phototoxicities determined by CCK-8 assay of (**a**) HeLa and (**b**) NIH3T3 cells treated with each sample (equivalent Ce6 10 μg/mL) and free Ce6 (10 μg/mL) under environment with CoCl_2_ or without CoCl_2_ for 2 h with light irradiation (for 10 min at light intensity of 5.2 mW/cm^2^ using a 670 nm laser source) (n = 7, as multiple experiments, ** *p* < 0.01 compared to free Ce6).

Figure 6a shows the in vitro antitumor efficacy of the dHA-Azo-Ce6 particles in HeLa tumor cells. Under the hypoxia condition, the accelerated Ce6 release of the dHA-Azo-Ce6 particles (Figure 3a) seemed to elevate singlet oxygen generation under light irradiation (Figure 3c). As a result, the dHA-Azo-Ce6 particles enhanced antitumor activity against CoCl_2_–treated HeLa tumor cells. However, another sample group (dHA-Ce6 particles and free Ce6), which did not respond to the hypoxia condition, showed no change in the rate of HeLa tumor cell death. In addition, all samples exhibited no significant difference in NIH3T3 cell viability regardless of whether or not they were treated with CoCl_2_ (Figure 6b). We also confirmed that there was no significant toxicity in the particles without light irradiation (Figure S2). Overall, these results suggest that the dHA-Azo-Ce6 particles are highly effective in eliminating tumor cells under hypoxia and light irradiation. In addition, in order to expect excellent in vivo antitumor effects, it will be necessary to develop a light source in the form of an ultra-fine probe that can provide light deep into the tumor.

## 4. Conclusions

Tumor hypoxia is recognized as an important feature of malignant tumors, and attempts to target this hypoxia are ongoing [1,2,3,4,5,6]. In this study, we successfully fabricated ultra-small dHA-Azo-Ce6 particles. The experimental results demonstrated that the dHA-Azo-Ce6 particles could accelerate antitumor drug release due to the Azo linker (–N=N–) cleavage in a hypoxic environment, increasing phototoxicity in HeLa tumor cells. We expect that these ultra-small particles are effective in treating hypoxia tumors. In addition, the therapeutic potential of dHA-Azo-Ce6 must be further explored through in vivo animal tests in the future.

## Data Availability

Not applicable.

## Supplementary Materials

The following supporting information can be downloaded at: https://www.mdpi.com/article/10.3390/pharmaceutics14050928/s1, Figure S1: ^1^H-NMR peaks of dHA-Azo-Ce6 treated with 10 mM Na_2_S_2_O_4_ for 2 h; Figure S2: In vitro cell viability determined by a CCK-8 assay of (a) HeLa and (b) NIH-3T3 cells treated with each sample (1–100 μg/mL) for 24 h without light irradiation.
