# Supplementary material for: Hypoxia-Responsive Azobenzene-Linked Hyaluronate Dot Particles for Photodynamic Tumor Therapy
Source: Pharmaceutics. 2022 Apr 24;14(5):928. doi: 10.3390/pharmaceutics14050928 (PMC9142920; doi:10.3390/pharmaceutics14050928)
Supplement: Supplementary file 1 [file pharmaceutics-14-00928-s001.zip › pharmaceutics-1700034-supplementary.pdf]

pharmaceutics

MDPI

Supplementary Materials: Hypoxia-Responsive Azobenzene-Linked Hyaluronate Dot Particles for Photodynamic Tumor Therapy

Sohyeon Lee, Yoonyoung Kim, and Eun Seong Lee

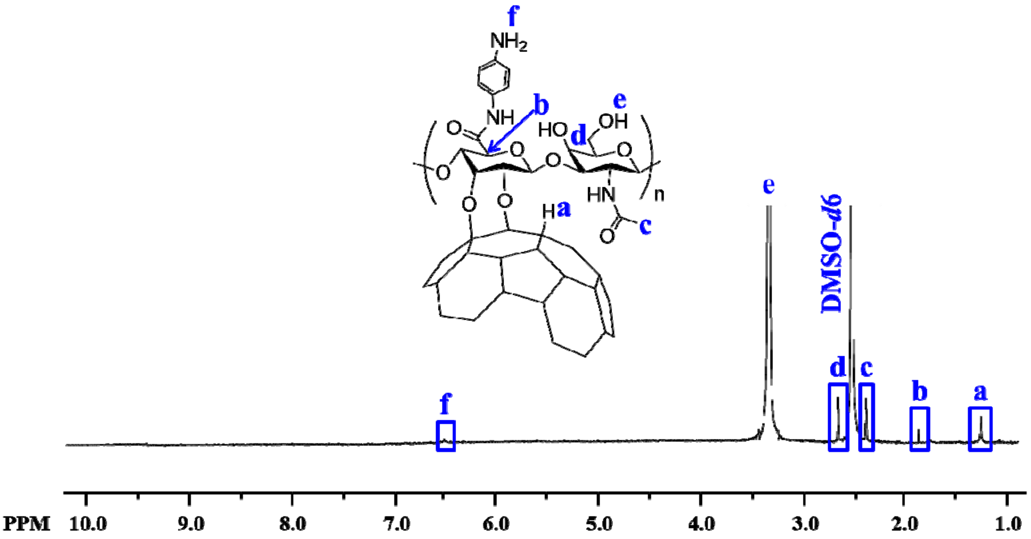

Figure S1. ^{1}H-NMR peaks of dHA-Azo-Ce6 treated with 10 mM $Na_2S_2O_4$ for 2 h.

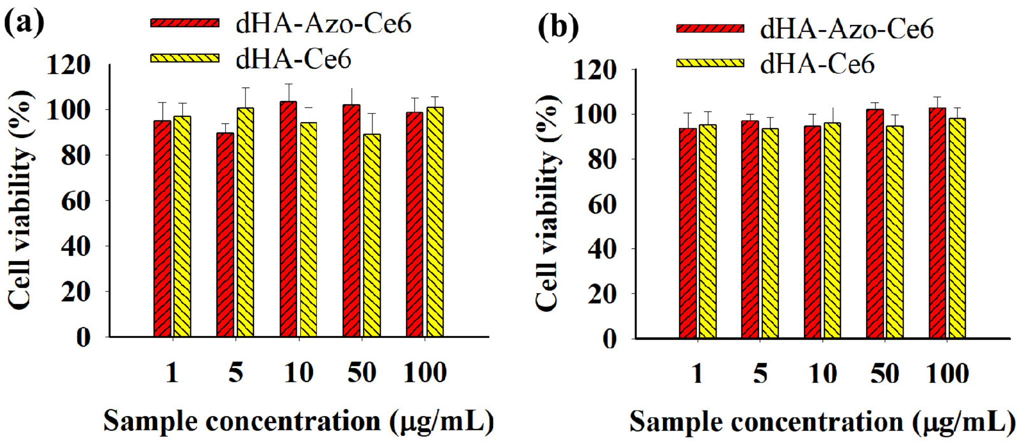

Figure S2. In vitro cell viability determined by a CCK-8 assay of (**a**) HeLa and (**b**) NIH-3T3 cells treated with each sample (1-100 µg/mL) for 24 h without light irradiation.
